# Thank You for Hearing My Voice – Listening to Women Combat Veterans in the United States and Israeli Militaries

**DOI:** 10.3389/fpsyg.2021.769123

**Published:** 2021-12-06

**Authors:** Shir Daphna-Tekoah, Ayelet Harel-Shalev, Ilan Harpaz-Rotem

**Affiliations:** ^1^Ashkelon Academic College, Ashkelon, Israel; ^2^Kaplan Medical Center, Rehovot, Israel; ^3^Conflict Management and Resolution Program, Department of Politics and Government, Ben-Gurion University of the Negev, Beersheba, Israel; ^4^Departments of Psychiatry and Psychology, Yale University, New Haven, CT, United States; ^5^National Center for PTSD, VA Connecticut Healthcare System, West Haven, CT, United States

**Keywords:** veterans, military, women in combat, trauma, transition, war, violence, military sexual trauma (MST)

## Abstract

The military service of combat soldiers may pose many threats to their well being and often take a toll on body and mind, influencing the physical and emotional make-up of combatants and veterans. The current study aims to enhance our knowledge about the combat experiences and the challenges that female soldiers face both during and after their service. The study is based on qualitative methods and narrative analysis of in-depth semi-structured personal interviews with twenty military veterans. It aims to analyze the narratives of American and Israeli female combat soldiers regarding their military service, with emphasis on the soldiers’ descriptions, in their own words, about their difficulties, challenges, coping and successes during their service and transition to civilian life. A recurring theme in the interviews with the veterans of both militaries was the need to be heard and the fact that societies, therapists, and military institutions do not always truly listen to female veterans’ experiences and are not really interested in what actually ails them. Our research suggests that conventional methods used in research relating to veterans might at times be inadequate, because the inherent categorization might abstract, pathologize, and fragment a wide array of soldiers’ modes of post-combat being. Moreover, female veterans’ voices will not be fully heard unless we allow them to be active participants in generating knowledge about themselves.

## Introduction

In April 2021, on Israel’s Memorial Day, combat veteran Itzik Saidyan set himself alight in front of the offices of the Rehabilitation Department of Israel’s Defense Ministry (Kubovich and Peleg, 2021). Following this shocking and devastating protest about ill treatment, many veterans, male and female alike, shared their painful memories with the public at large, and the issues raised by them prompted an intense public debate in Israel (Kubovich and Peleg, 2021). With the ‘Saaidian affair’ remaining in the media spotlight, veterans in Israel continue to speak out about their experiences in the battlefield and the impact of those experiences on their lives. Women veterans have added another dimension to this protest by revealing their double battle, one to actually carry out their military duties (with possible exposure to combat trauma) (Harel-Shalev and Daphna-Tekoah, 2020) and the other to integrate into a masculine military environment. Incidents like the one described above are not unique to Israel, and, sadly, situations of extreme distress among veterans, including suicide attempts, are not uncommon. In the United States, for example, each day sees about 18 veterans committing suicide (US Department of Veterans’ Affairs, 2020).

The continuously expanding body of knowledge on the implications of military service and armed conflicts for the lives of men and women combat soldiers spans a range of disciplines from psychology and other health sciences through critical security and military studies to political science and international relations. These disciplines both embody and reflect the events that influence the physical and emotional well-being of combat soldiers and veterans—men and women (Solomon and Flum, 1988; Harpaz-Rotem and Rosenheck, 2011; Daphna-Tekoah and Harel-Shalev, 2014; Rozanova et al., 2016; Grimell, 2018a). Just as the literature on veterans lies at an interdisciplinary junction, so, too, do the veterans themselves constitute crossroad protagonists in the negotiation of relations between politics, the state, the military, and society. Since many veterans bear the mental and physical scars of war, they could, in fact, be considered as “living monuments” (Jordan, 2011) who confront domestic societies with the consequences of the wars that those very societies sent them to fight. The responsibility of these societies is therefore fundamental (Bulmer and Jackson, 2015, p. 27; Grimell, 2018b).

Military service, particularly the service of combat soldiers, may pose a variety of threats and hence may inflict damage on both body and mind, with the literature on combat trauma indicating that combat does indeed leave a lasting impression on individual’s minds (van der Kolk, 2012; Homan et al., 2019; Fogle et al., 2020; Harel-Shalev and Daphna-Tekoah, 2020). After military service, former military personnel may develop post-traumatic stress disorder (PTSD) or other related psychiatric conditions, which may impair their adaptation to a new post-military, civilian life (Grimell, 2018a, p. 193). These challenges involve physical, mental and moral elements (Shay, 1994; Grimell, 2018b; Molendijk, 2018; Grimell and Nilsson, 2020; p. 380). A study on United States combat veterans vs. non-combat veterans revealed that combat veterans are more than three times as likely to screen positive for lifetime PTSD (Thomas et al., 2017). To date, the experiences of men combatants have constituted the main focus of studies on the psychological trauma of combatants, including combat trauma, PTSD, and symptoms of distress following combat. In contrast, research on the combat trauma of women soldiers has been sidelined out of the mainstream of trauma studies, in that it deals mainly with military sexual trauma (MST) and its affects, thereby relegating women, once again, to the category of the victim or the powerless. In this respect, trauma studies are thus gender biased (Daphna-Tekoah and Harel-Shalev, 2017; Harel-Shalev and Daphna-Tekoah, 2020; Kubovich, 2021).

As we note in earlier research, within the larger debate on military conscription, the dominant gender images of war have been relatively fixed for centuries: men are the militarists, women are the pacifists and/or victims. Men are warriors marching into battle, whereas women allegedly march for peace (Elshtain, 1995). Moreover, men’s participation in armed conflicts is viewed as a necessary component of citizenship, ethnicity, and communal belonging, whereas women’s participation in armed conflicts is not generally interpreted in such terms (Cooke, 1993; Enloe, 2007). And when women are involved in the battlefield, their contribution is typically underestimated. Nonetheless, more and more women are beginning to occupy different roles in militaries worldwide, thereby posing various challenges for these institutions (Goldstein, 2001; Eichler, 2013, 2021; Badaró, 2015). To counteract some of the conventional wisdoms about war and militaries (Cooke, 1993, p. 177), we regarded it crucial to provide a forum for women veterans to be heard on the subject of women’s presence and engagement at the front and hence engaged in the study reported here.

Against the above background, we, as a multidisciplinary research team of social scientists in the fields of health, trauma and security studies, sought to gain insight into the various scars of war through listening to the ways in which female combat soldiers presented their experiences (including traumatic experiences) in the military when they viewed their service retrospectively and when they discussed the problems facing them several years after their release from service. While we were well aware of the importance of providing quantitative data about veterans and manifestations of harm to veterans’ minds and bodies, we were equally curious to learn, on a qualitative level, about the nature of the experiences of these women and the threats and difficulties that they faced—and are still facing. In this sense, we believe that we have made a start on opening the way to address the criticisms aimed at studies that focus on binary categories. Critical military studies, for example, suggest that much of the quantitative research about combat trauma routinely objectifies veterans and their experiences and engages in “diagnostic competition over soldiers’ psyches” (Howell, 2011, p. 115). In addition, conventional quantitative methods used in research relating to veterans, such as surveys and questionnaires, might at times be inadequate, because categories might abstract, pathologize, and fragment a “wide array of soldier’s modes of post-combat being” (Wool, 2013, p. 406) and impose specific diagnostic categories. They might also fail to allow the veterans’ community to generate its own research questions or participate in the interpretation of the data gathered about veterans (Bulmer and Jackson, 2015, p. 29). Such a failure would be in keeping with the suggestion of van der Kolk (2012) that without truly listening to veterans we are not able to comprehend what actually ails them.

As women now serve in combat in larger and larger numbers, we chose to bring the narratives of women military veterans from both the United States military and the Israel Defense Forces (IDF) to the forefront of research about the trauma of combat soldiers and the transition processes experienced by military veterans with the aim to generate additional knowledge about their perspectives and difficulties. Since veterans are situated at the intersection of the military, the polity, and society, we felt that their narratives could inform the literature on combat and traumatic events from the view point of their “in-between” positionality. Here it should be remembered that military veterans should not be considered as homogeneous group, since veterans from various geographical and social climates and different genders may experience combat and post-combat difficulties in different ways. We thus approached this research from a multidisciplinary perspective and with intellectual curiosity about veterans’ experiences, stories, and challenges rather than viewing veterans as a “setback to be fixed.” The aim of this study was to explore the main elements that preoccupy female military combat veterans of the Israeli and the United States militaries when they view their service retrospectively, with emphasis on the veterans’ descriptions, in their own words, about their difficulties and successes during their military service and their transition to civilian life. By listening carefully to their stories, we aimed to trace the main issues that trouble them and to evaluate these issues comparatively in these two militaries and societies and thereby to shed new light on their experiences. We paid equally close attention what the veterans told us and how they told it. Through this methodology, the stories and narratives of the women combat veterans have thus become the supporting data for our study’s conclusions but, perhaps more importantly, they also provide insight into their experiences and struggles (Bagby et al., 2015; Molendijk, 2018).

## Descriptions of the Contexts and Challenges Due to Military Service

### United States and Israeli Militaries

The United States military has relied on an all-volunteer force for nearly four decades, with 0.4% of the United States population being on active duty (Reynolds, 2018). Women represent about 16% of enlisted forces (CFR.org Editors, 2020) and constituted about 10% of the deployed force during recent conflicts (Murdoch et al., 2014). In contrast, the IDF relies on mandatory conscription for both men and women, and about 50% of citizens over the age of 18 years are enlisted (Ultra-Orthodox Jewish and most Arab-Palestinian citizens are exempt). Women represent 33% of the military personnel, with 9% of women soldiers holding combat positions. After a long struggle, all roles in the United States military have been opened to women, whereas not all combat roles are open to women in the IDF (Harel-Shalev and Daphna-Tekoah, 2020). As may be gleaned from the above introductory sentences, our aim in selecting these two militaries was to present examples from an all-volunteer military vs. a mandatory-service-based military. In the United States case, women’s presence in the military is limited, whereas in Israel the proportion of women serving in the military is very much higher. In addition, the nature and the length of the deployment in combat service are usually very different in the two countries: in the United States military (and in many other large militaries), soldiers may be deployed for several months or even a year at a time to locations far away from home, whereas in the IDF, soldiers are typically deployed relatively close to home and for periods of up to 3 or 4 weeks.

### Combat Exposure to Trauma and Conflict in a War Zone

There is no question that military service is high on the list of potentially traumatic experiences (PTE) and qualifies as a criterion for the diagnosis of PTSD according to DSM-5 (American Psychiatric Association [APA], 2013; Lander et al., 2019). Although there are indeed studies describing stress and the consequences of combat exposure among both United States (e.g., Whaley Eager, 2014; Doran et al., 2021) and IDF (e.g., Daphna-Tekoah and Harel-Shalev, 2017) women soldiers, and traumatic experiences are indeed frequent among soldiers, society tends to shy away from detailed stories about trauma and from digging into moral injury (Shay, 1994), preferring laundered language and statistics about combat trauma (Harel-Shalev and Daphna-Tekoah, 2020, p. 69). Moreover, soldiers’ experiences of war are often shrouded in secrecy, as—historically—war veterans rarely describe closely what happens in the battlefield. On the surface, there may appear to be good reasons for this lack of candor, such as shielding partners, the wider family, and communities from the horrors of war (Wertsch, 1991). Yet, drawing a veil over the atrocities of war by not talking about them should not be taken to mean that these experiences are forgotten—“out of sight” does not mean “out of mind.” This point is well expressed by van der Kolk (2014) when he describes the devastating consequences for combat veterans of living with trauma—and with stories they cannot share. These consequences may include profound isolation, shame, guilt, aggression or violence, amnesia, disassociation, flashbacks, and reenactment. Symptoms like these, van der Kolk observes, are often met with inappropriate mental health care and a lack of understanding from others. Equally troubling is the reluctance of some clinicians to delve too deeply into the experiences of returning veterans (van der Kolk, 2014; Carless and Douglas, 2016, p. 375). Therefore, veterans with post-traumatic stress often experience difficulties in trusting others—be they clinicians, physicians, therapists, or government officials—and in maintaining emotional intimacy with loved ones, in large part because they feel misunderstood and isolated (Shay, 1994; Usry, 2019). Therefore, truly listening to and creating trust among veterans is of the outmost importance.

### Sexual Assault and Sexual Harassment Among Combat Soldiers and Combat Support Soldiers

Studies show that, globally, women in the military are in double jeopardy. They face the conventional dangers of being killed or wounded by the enemy during combat, in accidents, or in other ways (Jeffreys, 2007), and they also face the danger of sexual violence and harassment from their colleagues. Such events have a marked influence of their health (Castro et al., 2015; Brownstone et al., 2018).

Sexual harassment is prevalent in the United States military (Whaley Eager, 2014; Moreau et al., 2020) and also occurs, but to a lesser extent, in the IDF (Lomsky-Feder and Sasson-Levy, 2019; Shoval, 2021). In practice, women in professional militaries suffer more sexual assaults (Lomsky-Feder and Sasson-Levy, 2019, p. 13), and women in non-combat roles are more prone to be assaulted. Differences between United States and Israeli combatants may be partly is explained by the differences in the nature of the deployment in the United States military and the IDF, as described above. Estimates of sexual assaults during military service in the United States range from 9.5 to 49% among women (Castro et al., 2015; Wilson, 2018; Moreau et al., 2020). In Israel, numbers of complaints of sexual assaults in the IDF vary between 900 and 1,950 cases per year, which is less than 3% of women in the military per year (The Association of Rape Crisis Centers in Israel [ARCCI], 2020), but one suspects that these numbers are higher in practice, as some cases are not reported. Nonetheless, as a result of the above publications, we have some idea of the extent of the phenomenon, but qualitative data about MST is lacking. This lacuna is partly due to the fact that conducting interviews about sexual assaults, in general, is extremely difficult (Johnstone, 2016). Although it is known that MST is prevalent, ethical and methodological challenges make research using qualitative methods to examine sexual assaults rare (in comparison to quantitative research). Yet, knowledge from narratives is crucial for the development of a comprehensive understanding of this phenomenon that goes beyond categorization and the statistics of the assault and categorization of the aftermath implications. A recent study (Cortina and Areguin, 2021), in particular, provides important insights about the necessity for qualitative research, in that it allows the reader to grasp the lived experience of harassment.

### Military-to-Civilian Transition

State interest in veterans and the experiences of veterans has varied historically and geographically, depending on national and social contexts (Burkhart and Hogan, 2015; Bulmer and Eichler, 2017). In Western countries, such as the United States, Canada and the United Kingdom, the transition from military to civilian life is now recognized as a key social concern. Western countries are thus increasingly trying to support military-to-civilian transition through a suite of services and benefits delivered by military, state, the third sector and private providers. As long ago as 2014, Ashcroft (2014, p. 7) argued that good transition is important for the country (the United Kingdom in this case), because having invested in the training of military personnel, good transition can “ensure that those individuals are in a position to be net contributors to society.” According to Bulmer and Eichler (2017), it is “clear that the transition from military to civilian life has become a core project of Western governments in the early twenty-first century.” Governments recognize this “project” as important not only to mitigate the effects of war and military service, but to ensure the broader legitimacy of the armed forces and thus continued recruitment and retention. This suggests that the transition to civilian life is integral to the production of military force (Pellegrino and Hoggan, 2015; Bulmer and Eichler, 2017). On the personal level, veterans and service personnel must navigate a complex cultural transition when moving between the two environments (Cooper et al., 2018), and difficulties in the transition of both men and women have been reported (Rozanova et al., 2016; Bulmer and Eichler, 2017). In addition to the immense challenges each veteran faces—from PTSD to physical injuries (Harpaz-Rotem and Rosenheck, 2011; Burkhart and Hogan, 2015) —the additional stress of transitioning from military to home and civilian life could be immense (Grimell, 2017; Lander et al., 2019).

The military-to-civilian transition differs somewhat in the two countries. In the United States, the transition from active duty to civilian life has been documented as constituting a significant challenge (Kamal, 2021), particularly since only a minority of the population serves in the military. Upon their release, most men veterans feel misunderstood and confused when re-entering civilian life, and women veterans even more so (Decker et al., 2013), particularly as a substantial percentage of women veterans suffer from higher unemployment rates than their male counterparts (Greer, 2017). In Israel, the military-to-civilian transition is influenced by an earlier transition in the opposite direction: the close and institutionalized link between the transition to adulthood and entry into military service, due to the mandatory draft, is one of the central social mechanisms that normalizes military service within the life course and makes it seem “natural” (Lomsky-Feder and Ben-Ari(eds), 1999). Similarly, the societal norms that have been established may ease, to some extent, the reverse transition, in particular, the fact that the majority of young adult Israelis, women and men alike, follow the similar path of military service–time out–tertiary education might serve as a supporting mechanism for transitioning veterans (Harel-Shalev and Daphna-Tekoah, 2020). Research about the transitioning of Israeli combat women to civilian life has indicated the transition to be stressful for some, particularly due to a loss of the sense of their significance and importance in participating in the most crucial of state endeavors (Lomsky-Feder and Sasson-Levy, 2019, p. 77). Other Israeli women veterans reported that they moved naturally – occasionally with some minor “shock” – into their civilian lives upon completing their mandatory service (for some, with the additional year of service for officers), but for all, a feeling of competency when moving to civilian life was evident (Lomsky-Feder and Sasson-Levy, 2019, p. 78).

Qualitative research about transition processes from military to civilian life is acknowledged to be appropriate and adequate when taking into account narrative and storytelling of veterans in the complex phase of transition (Lomsky-Feder, 2004; Grimell, 2017, 2018a,b). We therefore felt the methodology of listening to the narratives of transition of women from different societies and militaries to be a valid means of informing ourselves about these transition processes.

## Materials and Methods

### Feminist Narrative Analysis

Through narrative analysis, scholars can see the interviewees as characters that are constructed as navigating between dilemmas and as expressing their own agency (Bamberg, 2020, p. 262). Moreover, as Gilligan has suggested: “The creation of trust is essential to people’s ability and willingness to speak truthfully about their experience, and listening in a way that creates trust thus becomes integral to psychological inquiry” (Gilligan, 2015, p. 75). This notion is particularly relevant to the study of veterans who are reluctant to share their vulnerability and difficulties due to the fear of stigmatization (Harel-Shalev and Daphna-Tekoah, 2020). Narrative analysis thus provides inductive knowledge (non-hypothesis testing), allowing subjectivity and experience into research (Levitt et al., 2018; Bamberg, 2020). The above ideas encouraged us to choose personal interviews and narrative analysis as our research tools; more specifically, we chose to use feminist narrative analysis to enable us to trace both spoken and unheard voices regarding the interviewees’ personal perspectives in specific socio-political contexts. In applying this methodology, we “listen to the plot,” as Gilligan guides us, by listening closely to the narratives. According to Gilligan (2015, p. 71), this listening “directs the researcher’s attention to the landscape of the interview or text (who is there, who or what is missing, are there repeated words, salient themes, striking metaphors or symbols, emotional hot-spots, gaps, or ruptures) and to the stories that are told.” This methodology thus enables us to disaggregate the personal and the political and to challenge the often taken-for-granted concepts that preserve hegemonic and/or patriarchal power relations (Gilligan, 2015; Harel-Shalev and Daphna-Tekoah, 2016; Arnd-Linder et al., 2018; Thompson et al., 2018; Gilligan and Eddy, 2021; Vaandering and Reimer, 2021).

In keeping with feminist narrative research that seeks to uncover previously neglected or misunderstood worlds of experience, we thus used narrative analysis to process the material. Listening to the women’s narratives enable us to gain a deeper understanding of the various interpretations of agency, in line with the notion that there are various narratives of knowledge among women (Ackerly et al., 2006; Stern, 2006; Enloe, 2014; Fenster, 2016; Ackerly and True, 2018). In narrative analysis, scholars typically direct their research to working with narrative and on narrative (Bamberg, 2012). In working first with narrative, knowledge is constructed in a bottom-up direction. In the second phase, on narrative, scholars analyze the interviewees’ narratives, according to their theoretical framework and research questions, by paying special attention to the ways in which individuals conform to and confirm existing orders (Wibben, 2011). In that sense, the scholars distinguish the meta-level (talking about experiences) from the content-level (experiences themselves). On the policy level, data from narrative analysis of interviews with veterans in combat roles in the military could constitute evolving knowledge about veterans’ well-being, exposure to threats and challenges, and coping mechanisms, and could thus inform policy recommendations.

### Research Population

The participants were 20 female military veterans, 10 Americans and 10 Israelis, who had completed combat or combat support service in the United States military or the IDF. All participants had been released from their service several years before the interviews were conducted (up to 10 years). The rationale for including combat support soldiers derived from studies showing that combat support soldiers suffer from PTSD in similar percentages to combat soldiers (Daphna-Tekoah and Harel-Shalev, 2017). Thus, combat support soldiers were included in this study, since they had also witnessed extraordinary violence and had been exposed to battlefields and war zones. The United States veterans were recruited via flyers placed at a large VA Medical Centers and in the community. The Israeli veterans were recruited via social networks.

### Ethical Considerations

The research received the approval of the IRBs of the Conflict Management and Resolution Program, Ben-Gurion University of the Negev (2017-02) and at the VA CT Healthcare System. Two of the authors are therapists, and the interviewees were notified that if there were any symptoms of distress following the interview, they could contact the relevant author directly. The participants were also informed about the background of the project (i.e., a need to gain knowledge about the process of military experiences and challenges, including transition to civil lives), the purpose of the project, the measures to maintain anonymity, and other formalities.

### The Interview Design

The interviews of the United States veterans were held in English, and those with the Israeli veterans, in Hebrew. The interviewers were of the same nationality and culture as the interviewees (American interviewer for American interviewees, and Israeli interviewers for Israeli interviewees). Semi-structured interviews, lasting 1–2 h were conducted with each participant. The interviewees were asked open questions about their deployment, their service, their exposure to violence and armed conflicts, their achievements and their lives after concluding their military service. They were all asked the same questions. To assure confidentiality, each veteran was identified by a pseudonym. With the participants’ consent, each interview was audio-taped and later transcribed verbatim.

### Narrative Analysis

Narrative analysis was then applied to the interview transcripts (Wibben, 2010; Caddick, 2011). Each researcher listened separately to the audio recordings and read the transcripts of the interviews. In listening attentively to the narratives, the researchers’ aim was to learn about the combatants’ experiences and insecurities and to unravel the unique ways in which they formed meaning in their own experiences in an environment defined by violence, constant threats, high-risk situations, and armed conflicts. The researchers and the research assistant, all fluent in Hebrew and English, also analyzed the data separately. They read the entire transcripts, made tentative interpretations, and marked the central ideas scattered throughout the narratives. The interviews that were held in Hebrew were analyzed in Hebrew and the interviews of the United States soldiers were analyzed in English. Then, the researchers and the research assistant met to discuss the findings and their responses to the interviews. They integrated the interpretative analysis of the dominant topics that were brought in the interviews and compared their conceptualization and analysis of the findings. The researchers then continued to theorize the interpretations and re-evaluate the findings to re-assess the main narratives that emerged in the interviews (Lev-Wiesel, 2007). In preparation for the publication of the article, several quotes from the Hebrew transcripts were translated into English.

## Results

The contexts and narratives necessarily converge into each other and are not isolated one from the other (Stern, 2005), but several important findings may be summarized: While listening to the plot of the interviews, we revealed what Gilligan termed as “the landscape” of the interviews, combining the personal and the political (Gilligan, 2015; Harel-Shalev and Daphna-Tekoah, 2016). Salient themes were exposed in the stories that were told. Three themes were dominant in the soldiers’ narratives—combat trauma, MST, and the transition to civilian life, including the lack of understanding of – and real listening to – their experiences (by society and the relevant state institutions). These themes appeared in a socio-political context in which the soldiers emphasized their capabilities and their struggles to fit in within the military in traditionally masculine roles. Before moving on to the main themes revealed in the interviews, this section briefly sets the scene – as Gilligan (2015) suggested regarding implementation of feminist narrative analysis – by presenting the settings of the participants’ experiences in which they expose their everyday military lives. In ’setting the scene,’ it is indispensable to note that we acknowledged and revealed, while listening to their stories, that they are experiencing all these aspects of the military service as combatants while struggling to integrate into these roles.

### The Battle to Integrate and to Prove Themselves

The interviews were taken place in a socio-political context in which women are included in combat roles in militaries worldwide for a few decades, and still, the findings emphasize women’s constant and ongoing struggle to prove themselves worthy of serving in combat roles both in terms of personal capabilities and of wider gender struggles. It was evident in both the American and Israeli narratives – as the women described in detail their actions and capabilities – that most of the women had faced a struggle to prove themselves. For example:

Olivia (United States military), who served in Kabul, Afghanistan, stated:

The females have to try harder to be better and stronger and faster than the males just to be considered even equal.

Harper (United States military) was even more direct in saying:

The biggest difficulty is that I didn’t have a penis; my male counterparts treated me like garbage.

Emma (United States military), who served in Kuwait, elaborated on the challenges facing a woman in combat:

I think in the military, in general, it was just more males than females. I think it was harder because you, as a female, you always have to kind of prove yourself to say that you can do the job, just because you’re not a male you can still do it regardless. You can still do the pushups. You can still do the run. You can still do the sit ups. You can still make rank. You could still push yourself. You can still go out there and complete these different obstacles as well as the males. I think that was the biggest thing…. I was proving myself there and here, ‘cause I had to prove to them that I could make it, regardless of what people were saying… the happiest moment for me in my career as a soldier after everything I went through in the military, I made it all the way to the sergeant.

In the same manner, their Israeli peers, tackled similar challenges. For example, Suzanna (IDF) emphasized the challenges of women in combat roles in the IDF:

It’s not only that I had to overcome these obstacles and courses, and to go through the navigation training, I had to prove that I could handle that well; I had to prove that I could make it, physically and mentally. Physically, I was tiny. What you need to take on your back weighs half your body weight. For a man – it is quite obvious that he can be a combatant, since being a combatant for a man it is not something out of the ordinary here [in Israel]. I felt very weird … I was really tiny, it was more difficult for me than for the rest of them.… And yet, I just loved it …. Physically, you’re not like a man, and you need to prove yourself ….

Zipora (IDF) affirmed a similar state of mind:

Still, you feel that in some way you need to prove yourself more than others [more than men]; since you are a woman in this place … this thought of how they will accept a woman in this place. You need to be very tough, to be very strict about every detail.

Christina (IDF) described her combat service as very complex and demanding, and added:

Both for ourselves and for others, we had to prove that we are worthy and deserve this role and we are strong and capable, they were watching us – we had to do everything perfectly, and not screw up. If you have to climb a wall, you climb a wall, and if you need to climb with a rope, you climb with a rope. If we don’t succeed, it will give them ammunition.

These above-described elements of the women veterans’ need to prove themselves and to show that they were equal to – and even better than – combat men, were frequently expressed, together with full descriptions of their capabilities and abilities. Although the socio-political context of the service derives from different societies and different militaries – volunteer versus mandatory conscription – their struggles to integrate, and to prove themselves worthy, were evident and dominant. Moreover, along with their description of their actions during service, we traced a strong sense of agency—an ability to choose and to overcome obstacles. Frequently mentioned in their stories and narratives was a sense of the ability to choose, to act, and to be good at the job.

Evelyn (United States military) described her challenging roles as combat medic and as a pilot:

Well, I mean I’ve done a lot.…I really loved being a medic…. I gained a lot of experience that played into my civilian life… being a pilot was so cool, flying helicopters. So, that had just a big impact on, like, [my] self-confidence. But yeah, I guess the deployments were probably like the most impactful…. I’ve been in a lot of leadership positions…. That was the big empowering things, because I went to officer school. I flew helicopters. I did all sorts of like big boy stuff, but it was always that. That was always really what made me feel good.

Evelyn was satisfied with her functioning and was gratified to gave been given the opportunity to do “men’s roles” in the military. Charlotte (United States military) talked about her abilities and satisfaction of serving in a very difficult role and under difficult conditions in her role in the army in Iraq:

I can do that, I get to do that, the whole physical activities… and I think that’s – that was very rewarding and empowering.

Similarly, the IDF soldiers discussed their abilities and assertiveness after fulfilling challenging roles, as well as the pride and satisfaction of being assigned to so-called “masculine roles”. Yulia (IDF) explained:

The major chose me as his radio operational officer in the field, he chose me and not others, there is a lot to it. You feel that you are empowered, you learn things about yourself, that you can be trusted, that others believe in you; it was right for me.

Britney (IDF) added insight into the unique position of a woman in a masculine role, and the admiration others felt for her role:

I carried a missile launcher connected to my M16 rifle. The orders are that you can’t leave it on the base, so I would take it home on the weekends. When people look at you… I can compare it to a woman with a very big cleavage … you see guys looking at me and saying to one another ’wow, look at her missile launcher’…. It’s an honor to be in the role of operator of a missile launcher, and when a woman walks around with it – it’s not ordinary. I remember a bunch of male combatants looking at me at the central bus station, and saying ‘wow! look at her.’

In the narratives of veterans from both militaries, the gratitude for the ability to serve in these roles and the satisfaction derived from them were evident in all interviews. Along with the sense of agency, the women were aware of their indispensable added value, and ability to act and to fulfill their mission in the best way that they could. It was also evident that many years after women were first incorporated into combat roles, they still feel grateful to serve in these roles. After introducing the above narratives relating to the women’s struggle to fit in and the satisfaction they derived from their abilities, we can now discuss the three main themes that emerged from listening to the plot of the narratives.

### Exposure to Traumatic Events and Combat Trauma

The analysis indicates that most veterans were exposed to at least some combat violence, regardless of their assigned duties. When asked to describe their primary duties, only about 50% of veterans reported that they had been assigned completely or mainly to combat duty, yet all reported moderate to heavy exposure to combat violence while serving. The scope of the traumatic events was varied, and several representative narratives that emphasize the trauma of the women’s experiences are presented below. Listening to the narratives and re-reading the transcripts about the veterans’ military service enabled us to grasp their experiences and to obtain a strong sense of what it means to be in surroundings of war and armed conflicts.

Camila (United States military) stated:

The war…it was…. The only thing that scared me so bad, was…. I was extremely scared for the whole six and a half months of SCUD missiles [in Iraq]…. So, we saw the SCUD missiles, the alarms went off for chemicals. We had to get into our MOPP gear and go…down to underground bunkers.

Indeed, the combatants were often exposed to danger, stress, and injury. Charlotte (United States military) reported:

I almost died myself, so I – it was close, very close…. And that was very significant. And … because this very significant person [her commander] was killed in action…. it was just a shock to me.… I still don’t believe it that he’s gone and it’s been long time ago.

The narratives of Camila and Charlotte indicated fear for themselves, but other narratives of traumatic events related to death or injury of peers. Evelyn (United States military) related:

I mean, of course, working as a medic there was a lot of traumatic stuff that you saw, so, sort of like rationalizing that and working with people that were sick, that were injured, and seeing… those immediate life changes, a lot of that was, it was really crazy.

Another experience of the horrors of war and violence during insurgency, was expressed by Harper (United States military):

The most influential experience ever in my life, ever, was the bombing of the United Nations building in Iraq; this had a lasting impact on my entire life. I can’t get it out of my head. I can’t make it better. I can’t make it stop replaying it in my dreams. Do you know what I mean? This was on my first deployment in Baghdad. I had already seen terrible things, terrible things, but this took the cake of terrible. The people that were bombed were just there to do humanitarian work. All they wanted to do was help the people of Iraq, mainly Baghdad, and they were blown away, and this to me, was an immense tragedy.

Similarly, their Israeli peers expressed parallel experiences of exposure to death, injury, stress and direct combat. Reut (IDF), who served as an operations sergeant on the Gaza front, described her response to a traumatic event during her service, and then reflected on the need to be detached as well as on the need to subsequently reconnect.

I arrived as an operations sergeant in the operations room near Gaza. During my second or third shift, a mortar bomb fell in the base. The noise was incredible…and then straight away that mortar bomb…[exploded] and the noise…just…the noise completely shocked me. It was the first time I had personally confronted anything like this…. It was the first time I had to confront something as stressful as this, and I was stressed out; under extreme stress…. I had some horrible shifts in that operations room…there was a time when all the biggest incidents happened to us. But when it happens…you detach yourself. You detach yourself not from emotions but from processing what happens. You just do your work… Afterward, you digest and understand what happened.

Reut mentioned how she encountered the fear of death and the stress of armed conflict for the first time. She was not prepared to experience such feelings and had to detach herself. Often, the veterans felt embarrassed and ashamed to admit their vulnerabilities and efforts to cope with trauma. Some of them felt that it is illegitimate to express these feelings.

Another example that gives insight into the veterans’ experiences is embodied by Betty (IDF), who was released from her service, having been diagnosed with PTSD, and was fighting to receive recognition from the Defense Ministry’s Rehabilitation Department. When asked about the difficulties of her military service, Betty described the adverse health effects of trauma:

You ask me if it was difficult…. If it was difficult to run? If it was difficult to jump or roll or to lie down in an ambush? No, it wasn’t. It wasn’t difficult to command a troop. These are the things that made me calm. The daily military life was stressful. The patrolling [on the border]…you are by yourself…sitting in this post for 4 h…it [the danger] is in your head and one thing leads to another. The emotional stress caused me physical injury, such as stomach aches and hyperventilation. It comes from nowhere, from my psyche.

Betty described the effects of the persistent threat that pervaded her everyday life in the military, serving on an active border and bearing huge responsibility. She was exposed to the threat of injury in every routine activity required by her role in field security and border security. The literature does indeed acknowledge the ways in which trauma is a burden on the psyche and the body, including weakening of the immune system and triggering the development of illnesses (Scaer, 2014), just as Betty had described.

Alexandra (IDF), when asked about difficulties during her military service, was not reluctant to admit her emotional vulnerability and the physical and mental effects of her exposure to stress, fear, and traumatic events:

I had a meaningful service and as a combat medic I did an excellent job, but…I remember fear…There were noises as if there were people digging in tunnels underneath our base, and the [men] soldiers told us: ’there is nothing here, relax’; now everyone knows it was true, they were actually digging…. I slept next to my weapon…there was a sense of fear…. Later, one of the women combat-support soldiers got killed…. Even long after I was released [from the military], I was very sensitive to loud noises and voices, after all these missiles and shootings. It was where the alarm doesn’t give you enough time to hide or go into the shelter, there is an alarm and an immediate boom (rocket falling).

One of the most troubling elements of Alexandra’s trauma was the fact that her evaluation of danger and her risk assessment of terrorist activities was disregarded. Overall, all the combat soldiers and combat-support soldiers, in both militaries, were exposed to traumatic events – including injury to themselves and to others – and remembered them in detail, long after their release from the military. Attentive listening to the detailed narratives of the combatants does indeed provide vivid evidence of combat trauma and coping. But the listeners, too, are people, and listening to stories of traumatic experiences of female combat soldiers can be difficult and even devastating. Nonetheless, scholars of trauma have an obligation to listen closely to the detailed narratives and acknowledge them and should not be reluctant to listen.

### Sexual Harassment and Sexual Assaults of Combat Women

The study revealed a substantial difference between the cases of sexual harassment in the two militaries. The narratives of MST of United States soldiers were frequent, and the severity of the assaults described by the combatants was extreme. The cases of MST reported by the Israeli combatants were rare and less severe. Below are representative narratives from their interviews:

Elizabeth (United States military) who served in Iraq, said:

The male soldiers were just like losing their minds. On other camps we heard about it too. Like, there was like a rapist who was waiting and attacking women and stuff on the other camp. Until like one chick like caught him and stuff….

Elizabeth’s description was not an exception, MST was dominant in the narratives of the American veterans about the nature of their service. The soldiers reported that MST was very frequent and that they and their peers suffered from this phenomenon; for instance, Isabella (United States military), a sniper, shared:

As a commander, I had a soldier who was sexually assaulted multiple times.

While Elizabeth and Isabella talked about assaults of others, Olivia (United States military), who served in Kabul, Afghanistan, shared her own sexual assault, within an environment that allowed these incidents to happen, both to women and to men:

Apparently, that was a big thing and most of the guys that I was over there with had a hard time dealing with females being in their unit because they were deployed, they had spouses or whatever back home that they didn’t get to see and apparently sex is a huge thing for guys more than females apparently and yeah, there were some issues. I was drugged and raped while we were over there by one of our troops…. I told the chaplain, I did not want to report it officially because it would have made it harder for the other females in the unit, as well as myself because I was pretty much the only person that could go out on combat missions because everybody else was either broken or couldn’t wear their battle rattle or whatever. So it would have made it impossible for them to succeed in their mission. But I did tell the chaplain that if this kid says anything to me whatsoever, I’m going to beat him to death and the Chaplin said he would hold him down for me. And then I found out like 4 weeks later or so that I was pregnant. And I told the chaplain what was going on, and then my grandmother passed away and he successfully got me emergency leave to come home. I came home, went to Planned Parenthood and had it taken care of. And the next day I was on a plane back to Afghanistan.

From Olivia’s narrative, we can realize how she justified the violent acts of some of the male combat men, since sex was a big deal for them during the long deployments. She also exposed how the system allowed her not to complain formally and to keep it as a secret within the unit, since she didn’t want to hurt the other women in the unit. She felt responsible for the representation of women in the battlefield. Olivia continued and explained about sexual assaults of men as well:

I think the guys had a hard time as well because I do know a few of them…that were raped while they were in the towers. And that was really hard because it’s hard for a guy to tell a female that they were sexually assaulted. It’s hard for a guy to tell anybody that because they feel victimized and vulnerable. But it happens.

She exposed how men are allegedly supposed to show less vulnerability and hence how these situations are extremely difficult for them as well. In her narrative, she described her rapist as a ‘kid’ and referred with empathy to male soldiers around her, saying how they, too, were often victims.

Emma (United States military) also shared her own painful experience of being assaulted and raped:

I was a victim of sexual harassment…. But I didn’t really—I don’t really see that in the beginning…. I didn’t really see it as sexual harassment. He was like that with me and my friends. I never really said nothing, because we were going to be deployed, and we have to be around him this whole time, and like he was a staff sergeant, and this was before I got my rank; even when I got my rank, he still outranked both of us…how can you say something about somebody that outranked you? He came from a another unit, but he deployed with us. So, who was really going to believe us?…. When you’re in a reserve unit, you only meet these people once a month…we talked to each other about it. He is married. He shouldn’t be doing stuff like that, but after he left our unit, and we came back from deployment, he ended up raping another—like he ended up raping somebody from his unit, so he ended up going to jail and everything…. So, I felt like if I had said something, maybe that wouldn’t have happened to her.

Emma was describing a painful rape, she shared with us how she was silent about it. At the same time, defining it as “sexual harassment” while clearly it was a case of a severe assault. What emerges from the above narratives is a common attempt to silence these incidences and not to expose them on a personal level, on a unit level or on the military system level.

The findings revealed that the Israeli narratives presented quite a different picture to the United States narratives. In the narratives that we analyzed, verbal harassments were frequent, but assaults were rare.

Rakefet (IDF) said, in response to our question about harassment and assaults in the military:

There were couples, not harassments; I can’t remember anything specific; there were always inappropriate comments by a certain male, but not anything drastic.

Silan (IDF) commented:

I was never harassed in the military. I didn’t see harassments, I heard of the case of this brigadier general. I heard of it but didn’t know them closely. The military does not tolerate such things, and the limits are strict and clear. The Israeli military system demands much more, in terms of values, than any other institution that I know.

Rosa (IDF) said that she first experienced sexual harassment in the military only when she became a reservist:

In my reserve service there was a guy who harassed me, and I stopped it. I didn’t complain formally because he has a family and children, so [instead] I opened it up with my officers. I said: ‘You either move him or me, I won’t stay in a place where I don’t feel safe.’ They handled it perfectly. They moved him to a different unit. They understood. From my perspective, it was a meaningful and a good solution.

The narratives of sexual harassment of the Israeli women soldiers in our study suggest that the experience of verbal harassments was frequent and uncomfortable and that the women typically handled such situations either on their own or with the support of their direct commanders. Yet, similarly to the United States narratives described above, these incidents, however, rare, were not treated through the appropriate legal mechanisms, and were settled within the unit.

Rosa continued:

In most of my mandatory service, I didn’t feel any sexual harassments. Sometimes men were hitting on me, and when I like it – then OK, and when I didn’t, I said no, and it was fine, as it should be. Most of my peers are my best friends until today. And they respected me and knew their limits.

Alexandra (IDF) recounted that mostly no assaults or harassment were documented, except for one incident:

There was a guy on the base, [he was] disgusting, and I was the only woman on the base. I was so naïve…. I was nice [to him], and he interpreted it as a reason to touch me. He was so disgusting. Since then, every time he came near me, I would go into the room and close the door…. Until I finally told him to fuck off. He was repulsive, he was a jerk, he went too far.

In summary, the narratives of the veterans indicated that verbal sexual harassments were frequent in the Israeli military and sexual assaults were relatively rare, although we can assume that some incidents had been silenced and not exposed. In the United States military, sexual assaults were much more frequent. The American veterans spoke about being insecure in instances in which men in their immediate surroundings acted violently or were abusive.

### Transition to Civilian Life

The experiences of combat women transitioning to civilian lives after their discharge from the military varied from interviewee to interviewee. While the overwhelming majority of interviewees expressed the feeling that their military service had been a beneficial and positive experience, nuances of difficulties could be read between the lines. Here, too, a difference was found between the two militaries. The transition for the American soldiers to civilian life was much more difficult, although they acknowledged that skills acquired in the military combat remain with them in civilian life:

Charlotte (United States military), managed to lead a normative civil life, to give birth to two daughters and pursue academic studies, yet she said:

Until today when I feel like I’m breaking into pieces [laughing]… I’m like, okay, I can do this, all that training that you had that sticks with you forever…, it [the military service] stays with you forever.

Charlotte admitted her vulnerability and laughed about it with embarrassment, yet she felt sense of agency due to her military training. Isabella (United States military) felt awkward about being a veteran and was not sure about her position among non-veteran students:

The actual transition out of military was to come here and start medical school. I’d say the toughest things has been like trying to figure out like when I tell people I’m a veteran or like how it even comes up in conversation. Is this something like I advertise? Because I can kind of blend in with the other students, and people who just assume I’m a lot younger than I am….

In contrast to the positions of Charlotte and Isabella, who underwent the transition to civilian life via their subsequent successful academic studies, other transition processes to civilian life were much more difficult and traumatic. For example, Elizabeth (United States military) said that during her transition to civilian life she did everything that she could in order to avoid people:

I think when I finally got out of the army, I was like, I’m not coming outside. I would really like going to grocery shop merely in gas stations, like anywhere I could go and grab milk and something. Or go to drive-thru’s.

Olivia (United States military) described additional difficulties in her transition process, including difficulties in academic studies:

I was discharged and then they gave me a rating of 90% disabled. I can’t relate to civilians. The lack of motivation, the lack of focus, the lack of determination, the lack of leadership, the lack of honor, integrity, you name it, it pisses me off. I worked as a restaurant manager for a very short period of time and yeah, I wanted to kill some of the people I worked with because it’s just ridiculous…. I’m in my first-year grad program. So it’s going all right, I guess. My first paper was a complete disaster. I’m not sleeping because of insomnia, so it’s really hard to focus.

Emma (United States military) added about her frustration with the system that is not helping her to adjust to civil life:

My transition was very hard. I didn’t have a lot of help. I didn’t have—it was just like okay. They told you about the services that they have. It was like they didn’t help me try to find a job. They didn’t help me try to get a job. They didn’t help me. They didn’t point me in the right direction. They didn’t assign somebody to me to help me get a job…. I didn’t want to talk about me being different now, because I wasn’t the same person. Life had—in the military had changed who I was, like it was a lot of different bad things…bad things changed who I was now. I wasn’t what I was before I went into the military. I wasn’t the same person. You can’t tell your friends here that, because they’re not going to understand.

Elizabeth, Olivia and Emma gave voice to the idea that the society that had sent them into battle and into military service was not doing enough to assist them in their civilian lives. In their interviews, they indirectly criticized the system for not taking responsibility for the consequences of the processes that had changed their lives, and called for attentive and proactive action to assist them to adjust to their civilian lives.

Similarly, Amelia (United States military) reported difficulties in her daily life:

I had a really hard time relating to people who didn’t come with me. I had a hard time driving. I would swerve when things were in the road and not even think about what the other cars around me—like it was just a hard time going under bridges…but things that still trigger me are fireworks, noises…. So, noises, I’m still very jumpy, and it may not even be someone’s voice, but like just a loud noise because we were constantly mortared.

Harper (United States military) further explained about her military afterlife:

Thank you for asking this. I don’t really know how to be a civilian, so what do I do? I go to college, because I don’t know how to be a civilian. So I was like, well, I’ll just go to college. I’ll finally do that, and I didn’t really do that. I mean, I excelled at going to college, but I didn’t really go to college well. I couldn’t handle being around people who were younger and so naïve. Oh man, I just couldn’t handle it. I don’t think I’ve ever really transitioned to being a civilian. I’ve not been able to hold down a job. I haven’t had a job since, really.

The interviewees exposed their vulnerabilities, difficulties, and pain in their struggle to cope with moving from being a soldier to becoming a civilian; they felt that they did not receive the appropriate tools or the appropriate guidance to successfully cope with all the difficulties of the transition and they felt alone in their struggles to cope.

In contrast to the United States military women, who are a minority in the society that they are serving, many Israelis serve in the military, and the fact that a large proportion of society goes through a similar process could be considered as a supporting mechanism, since military service is regarded as a normal step in “growing up.” The difference between the two societies was striking and was reflected in the narratives:

Ruhama (IDF) stated:

It is a bit shock, at the beginning, that you are not in this system, that you wake up every morning and there is the routine that you know what to do. Actually, I was happy to move on with my life since I was immigrated to Israel and the military was a part of it, but I always knew it is a part of life and then you move on to academic studying; this is another part of your life.

Betty (IDF) reported:

In the beginning, it was like a dream, I was happy to be at home, I wanted my family all around me, I didn’t have to go to the base for 3 weeks; no one was nagging me and telling me what to do. And people were asking – wow, were you released already? You were a combatant in Caracal [a coed infantry battalion in the IDF]? Wow!! Impressive, good job. And I didn’t really know what to say, because it was great, but I also was hurt emotionally and physically from the military.

Rosa (IDF) spoke about her transition:

I was released and immediately drafted to reserve service, so I was busy and the transition was quite smooth. The transition to civilian life was good. In the service as a combat commander I was responsible for 500 or 700 soldiers and it is a huge responsibility. I had such a complex and sensitive role, and then poof. You are at home. It took me a long time to learn about how to put things in proportion. In the military everything is super important; and in civilian life there are things that are less important. I was used to the fact that timing is sacred; I remember that I arrived at a job interview 30 min ahead of time and they asked me: ‘why are you here so early?’ It took me a while to understand how to understand the proportions and the priorities in civilian life, but I learned to get used to the fact that not everything that I do has top priority and national security importance. It is nice.

Some of the women were still coping with traumas at the time of the interviews and faced difficulties in their dealings with state institutions. It was thus not surprising that some of the narratives about transition to civilian life included references to frustration with the military system and its affiliated civil institutions, as well as complaints about that fact that the “system” did not really “get them right.”

Emma (United States military), an automated logistics specialist, who served in Kuwait, explained:

I receive mental health services here. I think that if people feel that they need it, they should get it. I don’t think that medication is always the best option…. I stopped coming for a long time, because—I mean the provider wasn’t—we didn’t—so I stopped coming for a long time….

Emma felt that her treatment was not adequate or helpful for her and it caused her to decide to quit her treatment. Similarly, Amelia (United States military), a medical specialist and pharmacy technician, who served in Iraq, discussed her experiences with counselling:

Well, I know like for myself it’s scary to go [to therapy], it’s scary to talk about the things that you want to avoid. So, I know in my experience, you know, the way that I deal with things is avoiding them, and then I go to counseling, and they want me to talk about my experiences, and then I’m sitting there thinking okay, well, I’m talking about this experience. How the heck is this going to help me? I mean, I feel like maybe there is a tool so, or even like with, even if they prescribe me something because I’m having this symptom, I’m like really like how’s that going to help me if you just put a Band-Aid on it like—I’m not looking to be medicated. I’m looking to find the solution so I don’t have to be medicated, and then so, I kind of feel at a loss. I think that I would jump to counseling more if I knew that they really had the tools that I needed for the situations I come up against… I think maybe even asking me what I think might help, so that I have to sit down and really think about what it is.

Harper (United States military) explained a problem of mistrust of therapists and health care services among veterans, who feel that the health care system is not really attentive and is often patronizing. For example, she emphasized the frequent problematics of prescription medication to veterans:

You just say some fancy word, and then I don’t know what that means. I think that gives a lot of veterans a lot of anxiety. Maybe they’re not going to take that medication, because they don’t know what that means… veterans have this touch of mistrust, like you’re going to give me a substance, and say here’s this fancy word thing, it’ll make you feel better. But what is it really going to do?

Harper concluded her narrative and interview by saying to her interviewer “thank you for hearing my voice,” indicating how important it was for her was to share her experiences and to feel that someone cares about her and her difficulties following her military service.

The above perspectives presented by United States veterans caused us to reflect about the solutions offered to veterans and led us think about the possibility of enhancing and promoting joint peer groups that could share their experiences together (we discuss this option below).

Among the Israeli veterans that was interviewed in this study, Betty was the only one who complained about the level of attentiveness in the part of the military system and the relevant offices, Betty (IDF) said:

When you asked me about the transition and the military system, it is really paradoxical for me, since on the one hand I really loved the service and I really wanted this combat role. I wanted to be a combatant in Caracal and I wanted a meaningful service, as all my male friends. I have great memories and I gained so much; but since I was wounded when I was released, I sensed that the system was not receptive and not understanding and I had to fight for recognition of my PTSD and my medical problems. That was really disappointing. So there are two sides to my story – on the one hand the service was really awesome, but on the other, disappointment at the lack of understanding and the lack of responsibility for my suffering. So, it is this and that combined.

Among the Israeli combat soldiers, each one said that the interview with us was the first time anyone had asked them about their experiences and their transition, and that they had not really shared the experiences with anyone but their peers. Betty was the only one of the ten who felt betrayed in a way, since the military acknowledged her medical problems only a few years after the end of her service and only after a long battle, so she felt she had not been heard.

In summary, as in previous research, the participants expressed appreciation for their training and pride in their abilities and in the opportunity to serve their country and to contribute to their society.

## Discussion

Military veterans should not be seen as a homogenous group, both within the group of veterans from each country and among veterans from the two different countries—their perspectives are diverse. Yet, some trends could be identified in their narratives regarding their sense of security and insecurity. As mentioned above, semi-structured interviews were carried out with the aim of allowing the respondents to give an account of their experiences in the military and their transition from military to civilian life in their own words in a flexible and dynamic manner (Smith and Osborn, 2007; Grimell, 2018a). During the interview process, the combatants discussed adjustment to life within the military, meaningful experiences and difficult experiences in the military, resettlement support, and adjustment issues upon discharge from the military. The recurring theme in most of the interviews in both militaries was that although the veterans were very proud of their abilities and achievements, they all experienced struggles to prove themselves worthy as women who were positioned in prestigious roles of combat and combat support. The interpretation of their experiences, narratives and stories should be understood in that context.

There appeared to be an overwhelming feeling on the part of the interviewees that their experiences in the military were not important or interesting to the military itself or to society at large. They sensed that they themselves acknowledged the importance of their roles and contribution to national security and to the protection of the state, but that the ‘system’ was not always appreciative and – worse still – not always attuned to their perspectives and needs. As in other studies (Brooks et al., 2016), several interviewees voiced frustration in enrolling in and understanding veterans’ health care systems, particularly regarding the recognition of disabilities and compensation. This was true for the Israeli narratives, but very much more so for the American narratives.

In both groups of veterans, the interviewees thanked us directly and indirectly for the opportunity to be heard and to share their stories. In the United States narratives, most interviewees voiced the opinion that their therapists were not really capable of understanding what they had been through and were therefore not fully equipped to undertake their care, since the therapists had no military experiences or personal experience of combat trauma (Atuel and Castro, 2018). In the Israeli narratives, most soldiers admitted that this was the first time they had shared their experiences in such a comprehensive manner and that they had not really been asked about the service or post-service periods of their lives. For most interviewees – in both militaries – the service had been meaningful, but at the same time it had exposed them to combat trauma on a regular basis. Indeed, they were exposed to it while trying to prove themselves adequate for their roles. Research has indicated that military cultural norms may stigmatize psychological injury as “weakness” and prevent individuals from seeking help (Stanley and Larsen, 2019); this outlook was indeed evident in the current research. This finding led us to think further that both scholars and interviewers who are working with veterans should be either have gone through similar processes, or – if not – should intimately learn the particularities of the military culture and military nuances to enable them to learn to be attentive listeners. A further interesting finding was the need to be heard, which connects the meta-level (talking about specific experiences) and the content-level (the experiences themselves). The female soldiers repeatedly mentioned that their experiences were not interesting to others or that they felt misunderstood by therapists and others. Mentoring programs of veterans could be beneficial, both in enabling the veterans’ agency and in normalizing their experiences.

The most marked differences in the narratives were those relating to sexual assaults in the military and to the military-to-civilian transition. In the United States military, sexual assaults are frequent, and the treatment of MST incidents includes many cover-ups that are harmful to those who have been assaulted. In contrast, in the Israeli group, descriptions of assaults were rare. However, in both groups, military sexual harassment was frequent. The low prevalence of sexual assaults in the IDF (3%) is surprising in comparison to the figures for the United States military, in which sexual assaults are as frequent as a pandemic. This difference might be explained by the fact that in professional armies, rates of assault are higher than those in mandatory service, since reporting often prompts retaliation. In addition, the nature of the deployment is different in the two armies, being much longer in the United States military. Nonetheless, more research is needed to explore these phenomena, and steps should be taken to create a safer environment for women in the militaries of both states.

As mentioned above, the transition to civilian life also differed in the two countries, with the transition in the United States being extremely traumatic for most interviewees. In contrast, the Israeli interviewees reported experiencing only minor shock; in particular, they did not feel isolated from society, since many of their friends and relatives were going through, or had gone through, a similar process (Friedman-Peleg and Bilu, 2011). This led us think about further steps that should be taken in both militaries in order to ease the transition, we present these ideas later in the article.

Let us now examine what can be gained from listening to soldiers’ authentic narratives (Hicks, 2011). Such narratives can shed light on veterans’ multidimensional experiences during their service, not merely as subjects that have experienced traumatic events, but particularly as those who have the agency and ability to function, to fight, and to feel a variety of emotions. In keeping with this idea, Gilligan (2015, p. 75) has warned researchers away from what she terms as “binning,” where binning is “taking someone’s words and sticking them into mental bins, [which] signifies a mode of listening that is not really listening but rather assimilating the experience of another to what one already believes. It is a way of asserting dominance and also an expression of disrespect.” Our aim was thus to trace the soldiers’ main narratives and to disaggregate them, while at the same time acknowledging the veterans’ complex, multidimensional and diverse experiences. Furthermore, Gilligan and Eddy (2017, 2021), in their recent study, indicate that health professionals have begun to research and to write about how doctors, nurses and therapists can listen to their patients more effectively, and this study thus forms part of this new corpus of research. It is our hope that we have managed to listen sufficiently closely to the veterans’ stories and that this research will alert societies, therapists, and military institutions to the fact that they are not always listening closely to veterans’ experiences and are not always really interested in what actually ails them.

While a number of studies examine the reasons why veterans defer care (e.g., Brooks et al., 2016), including stigmatization, this study offers another layer underlying this situation, namely, the perception on the part of the veterans that they are not being heard and not being truly understood or listened to. Doran et al. (2021), in listening to veterans in therapy, identified multiple barriers to treatment completion and provided insight into the veterans’ thoughts and feelings during the treatment protocol. As in other studies about veterans, the participants in our study, particularly those in the United States, indicated that they felt that the therapists did not fully understand their needs and concerns, since they had not experienced the military in any way. In Israel, the idea of combat service is known to all and experienced by many, so therapists and veterans do have a common background, but one interviewee said the military did not take responsibility for her injury. This was also true for the case of Saidyan that was mentioned at the beginning of this article.

The above findings indicate that several levels of listening are needed and that society ought to listen, since it sends its youth to the military and therefore is obligated to pay attention to the consequences of military service. Another layer of listening is related to the fact that the combat soldiers are exposed to difficult experiences and traumatic events, and even if they do not develop symptoms of distress, they deserve to be heard and to express their voices. Ashcroft (2014) stated that mainstream academic and policy research routinely objectifies veterans as “problems to be solved” rather than as a substantial part of the society that were defending. It seems that societies and states should be obligated to protect their veterans and to acknowledge the heavy toll of the fighting. As the current study suggests, just as the military invests effort in the training of combatants and in the selection process for assigning soldiers to particular roles, so is the military similarly obligated to make an effort to guide veterans back to civilian lives while taking care of their wellbeing.

Perhaps one of the solutions to the dilemmas raised in the narratives of the veterans could be found by focusing on detailed descriptions of traumatic experiences, challenges and difficulties, instead of moving directly to diagnosis of symptoms of distress, PTSD, and psychopathology. Moreover, it should be remembered that women who enter combat roles have to cope with both physical and mental difficulties, deriving from their exposure to life-threatening events, death, and other traumatic events. In addition, they have to cope with gender inequality and hegemonic masculinity in the military, as well as sexual harassments, and in some cases, abuse. We suggest that professional closed group discussions with women combatants who are about to be released from service should be held on a regular basis, not merely to identify PTSD, but also to allow the soldiers to share their feelings, emotions, traumas and experiences, thereby making them visible, heard and thanked for their contributions and actions. These group discussions could provide support for the soldiers by giving them a framework to feel that they are not different and not alone and could also allow therapists to identify vulnerabilities and risks for developing symptoms of distress. The framework could also be leveraged to acknowledge the female soldiers’ abilities and agency.

The current study has several limitations, most prominently the small sample size. Obtaining qualitative data is labor intensive and hence by its very nature necessitates smaller samples, but for drawing more definite and far-reaching conclusions it is necessary to conduct further studies of larger, more diverse, and more representative samples of veterans. Another limitation is the vast differences between the societies and the militaries that could also affect the differences in trends among female soldiers in both militaries. We chose specifically these two contextually different groups particularly in order to explore the similarities and the differences.

Despite its limitations, this qualitative study does provide a multifaceted analysis of the narratives of female combat and combat support soldiers who served in the United States and the Israeli militaries. In so doing, it attempts to avoid what may be termed as the “the danger of misrepresentation” that exists in some studies about veterans, because in speaking for or about others, one typically claims to have understood something about them (Caddick et al., 2017, p. 15). By bringing the detailed narratives of the soldiers to the forefront of the article and presenting them to our readers, we aim not to speak for the veterans but to offer their retrospective perspective of their service. In this way, we hope that by informing and extending the social conversation about veterans and veterans’ issues, this project will assist in easing their transition to civilian lives and provide tools for healthcare professionals.

## Conclusion

In this article, we have shown the main issues that the soldiers are required to cope with, what their most vivid memories are, and what topics were dominant in their military service and still prevail in their lives, years after their release. Two of the dominant recurring themes in the interviews with veterans of both militaries was the need to be heard and the fact that societies, therapists, and military institutions do not always truly listen to veterans’ experiences and are not interested in what actually ails them. Our research suggests that conventional methods used in research relating to veterans might at times be inadequate, because categorization can abstract, pathologize, and fragment a wide array of soldiers’ modes of post-combat being. We suggest adding more research frameworks that would allow the veterans to be active participants in generating knowledge about themselves. We further suggest activities that enhance their agency in their entrance back to civil life.

## Data Availability Statement

The original contributions presented in the study are included in the article/supplementary material, further inquiries can be directed to the corresponding author.

## Ethics Statement

The studies involving human participants were reviewed and approved by the IRBs of the Conflict Management and Resolution Program, Ben-Gurion University of the Negev (2017-02) and at the VA CT Healthcare System. The patients/participants provided their written informed consent to participate in this study.

## Author Contributions

All authors listed have made a substantial, direct, and intellectual contribution to the work, and approved it for publication. All authors contributed equally to this article.

## Conflict of Interest

The authors declare that the research was conducted in the absence of any commercial or financial relationships that could be construed as a potential conflict of interest.

## Publisher’s Note

All claims expressed in this article are solely those of the authors and do not necessarily represent those of their affiliated organizations, or those of the publisher, the editors and the reviewers. Any product that may be evaluated in this article, or claim that may be made by its manufacturer, is not guaranteed or endorsed by the publisher.
